# Bacteria-targeted fluorescence imaging of extracted osteosynthesis devices for rapid visualization of fracture-related infections

**DOI:** 10.1007/s00259-022-05695-y

**Published:** 2022-01-26

**Authors:** Marina López-Álvarez, Marjolein Heuker, Klaas A. Sjollema, Gooitzen M. van Dam, Jan Maarten van Dijl, Frank F. A. IJpma, Marleen van Oosten

**Affiliations:** 1 Department of Medical Microbiology, University of Groningen, University Medical Center Groningen, Hanzeplein 1, PO BOX 30001, 9700 RB Groningen, The Netherlands; 2 Department of Cell Biology, University of Groningen, University Medical Center Groningen, Groningen, The Netherlands; 3 Departments of Surgery, Nuclear Medicine and Molecular Imaging, Medical Imaging Center Groningen, University of Groningen, University Medical Center Groningen, Groningen, The Netherlands; 4 TRACER Europe B.V./AxelaRx, Groningen, The Netherlands; 5 Department of Surgery, Division of Trauma Surgery, University of Groningen, University Medical Center Groningen, Groningen, The Netherlands

**Keywords:** Fluorescence imaging, Bone fracture, Trauma, Bacteria, Infection, Vancomycin

## Abstract

**Purpose:**

Fracture-related infection (FRI) is a serious complication in orthopedic trauma surgery worldwide. Especially, the distinction of infection from sterile inflammation and the detection of low-grade infection are highly challenging. The objective of the present study was to obtain proof-of-principle for the use of bacteria-targeted fluorescence imaging to detect FRI on extracted osteosynthesis devices as a step-up towards real-time image-guided trauma surgery.

**Methods:**

Extracted osteosynthesis devices from 13 patients, who needed revision surgery after fracture treatment, were incubated with a near-infrared fluorescent tracer composed of the antibiotic vancomycin and the fluorophore IRDye800CW (i.e., vanco-800CW). Subsequently, the devices were imaged, and vanco-800CW fluorescence signals were correlated to the results of microbiological culturing and to bacterial growth upon replica plating of the imaged devices on blood agar.

**Results:**

Importantly, compared to culturing, the bacteria-targeted fluorescence imaging of extracted osteosynthesis devices with vanco-800CW allows for a prompt diagnosis of FRI, reducing the time-to-result from days to less than 30 min. Moreover, bacteria-targeted imaging can provide surgeons with real-time visual information on the presence and extent of infection.

**Conclusion:**

Here, we present the first clinical application of fluorescence imaging for the detection of FRI. We conclude that imaging with vanco-800CW can provide early, accurate, and real-time visual diagnostic information on FRI in the clinical setting, even in the case of low-grade infections.

**Graphical abstract:**

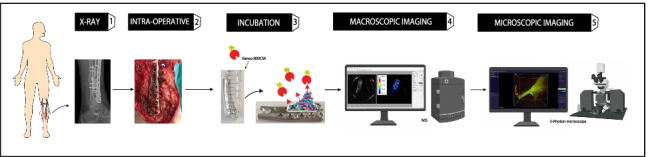

**Supplementary Information:**

The online version contains supplementary material available at 10.1007/s00259-022-05695-y.

## Introduction

FRI is a devastating complication after bone fracture treatment, resulting in severe patient morbidity and loss of quality of life. FRIs can be differentiated into (1) acute infections with clear signs of infection caused by highly pathogenic bacteria and (2) low-grade infections caused by less pathogenic bacteria. Low-grade infections often lack specific clinical symptoms, which complicates their diagnosis. Treatment of FRI usually consists of revision surgery, including debridement and removal of infected bone and osteosynthesis devices (e.g., plates, nails, and screws), combined with long-term antibiotic therapy [1, 2]. During surgery, samples are collected for culture to verify infections and identify causative microorganisms for specific antibiotic therapy [2–4]. A major challenge is a distinction between noninfected and infected areas and devices during surgery, especially in the case of low-grade infections. Surgeons rely on tactile and visual information to establish whether and to what extent wound areas are affected by the suspected infection. Further, a definitive preoperative diagnosis of FRI is often not possible in the case of low-grade infections due to the absence of unambiguous clinical symptoms of infection. In particular, the results from diagnostic modalities, such as white blood cell scintigraphy, fluorodeoxyglucose positron emission tomography (FDG-PET), or magnetic resonance imaging (MRI), are often inconclusive [3, 5, 6]. Therefore, a diagnostic imaging tool enabling early, accurate, and real-time visualization of the infecting bacteria themselves, rather than inflammatory responses elicited by the infection, would provide a solution to this clinical problem.

Bacteria-targeted optical (i.e., fluorescence) imaging is an upcoming clinical imaging technique with great potential for the visualization of infections [7]. In particular, a conjugate of the antibiotic vancomycin and the near-infrared fluorophore IRDye800CW (i.e., vanco-800CW) was previously identified as an effective bacteria-targeted optical tracer in preclinical infection imaging studies [8, 9]. Like vancomycin, vanco-800CW specifically targets the Gram-positive bacterial cell wall. Since FRIs are mostly caused by Gram-positive bacteria, such as *Staphylococcus aureus*, vanco-800CW seems particularly suited as a bacteria-targeted optical tracer for real-time diagnosis of FRI. Moreover, vanco-800CW efficiently binds Gram-positive bacterial biofilms as encountered on FRI [10].

The overarching objective of our present study was to obtain proof-of-principle for the use of bacteria-targeted fluorescence imaging to detect FRI on extracted osteosynthesis devices as a step-up towards real-time image-guided trauma surgery. The specific aim was to investigate whether vanco-800CW allows discrimination between infected and noninfected osteosynthesis devices and whether it can be applied to visualize bacterial biofilms in clinical practice. To this end, we investigated osteosynthesis devices from 13 patients, who needed revision surgery and extraction of plates, screws, and/or nails after an unsuccessful prior fracture treatment. In addition, we addressed the question of whether fluorescently labeled vancomycin allows the detection of vancomycin-resistant Gram-positive bacteria.

## Materials and methods

### Fluorescence imaging of extracted osteosynthesis devices

**Fig. 1:**
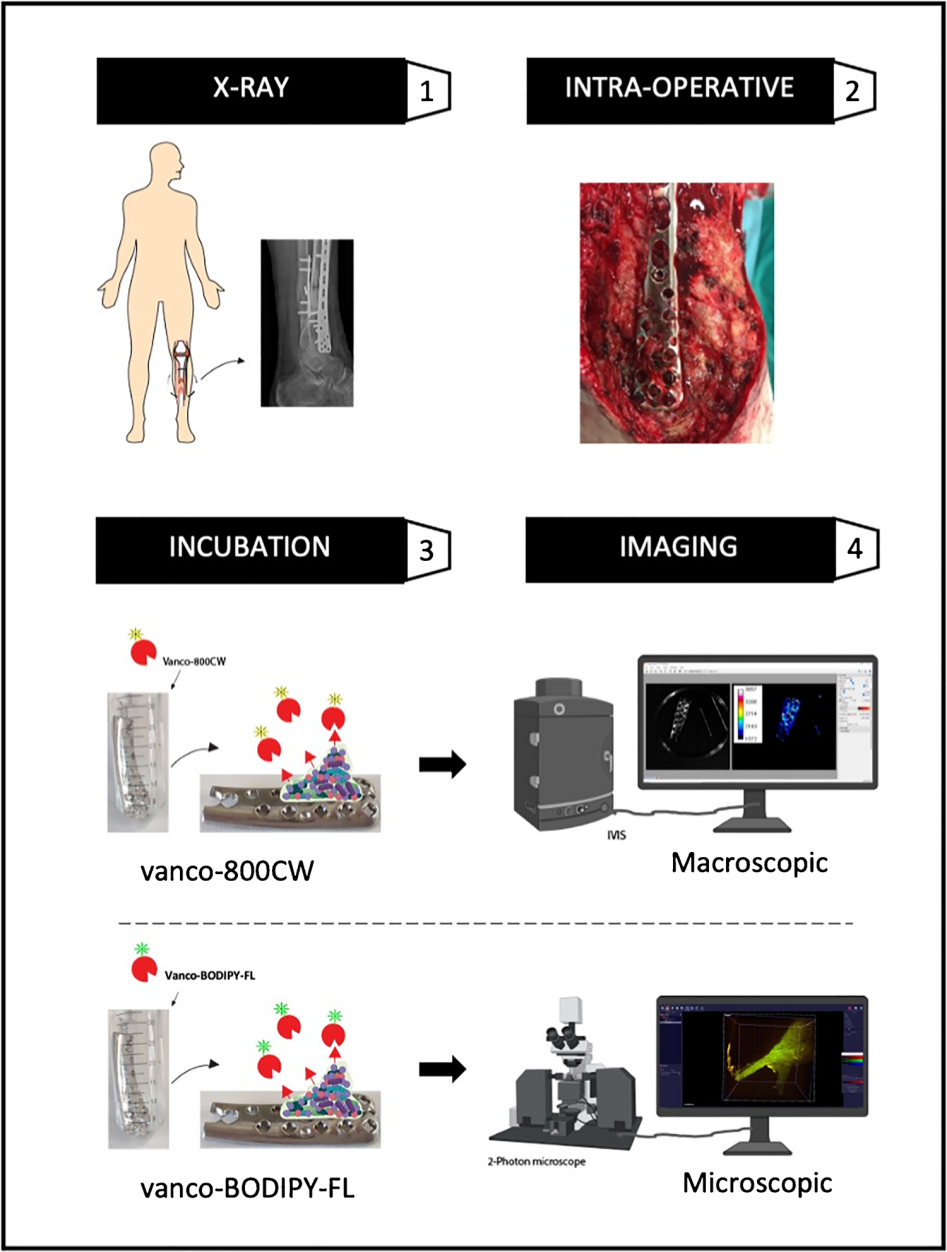
Schematic overview of the fluorescence imaging workflow. (**1**) Schematic representation of a patient with the clinical suspicion (i.e., local redness and swelling around the scars) of a fracture-related infection after surgical treatment for a distal tibia fracture. An X-ray image demonstrates the nonunion of the distal tibia. (**2**) Subsequently, plates and screws are extracted during revision surgery, and an intraoperative image is recorded to document whether signs of infection are detectable by the eye. (**3**) The extracted osteosynthesis materials are washed with PBS, incubated for 15 min with vanco-800CW or vanco-BODIPY FL, and washed twice with PBS to remove unbound vancomycin-based tracers. (**4**) Lastly, the extracted materials are imaged. Macroscopic imaging with an IVIS Lumina II imaging system and an intraoperative Explorer Air camera coupled to a closed-field imaging box (Vault) is performed to detect bacteria-specific fluorescence signals emitted by vanco-800CW. Microscopic imaging with a two-photon confocal laser scanning microscope is performed upon labeling of extracted materials with vanco-BODIPY FL to verify and visualize the presence of bacterial biofilms. Part of this figure was created with BioRender.com

Osteosynthesis devices extracted from 13 trauma patients were included in this study. During surgery, the removed plates were divided in two by using nippers. One-half of the plate was used for fluorescence imaging, and the other half was used for regular diagnostics (described in the following section). A number of extracted screws, all retrieved from the same surgical site, were used for regular microbial diagnostics and some for fluorescence imaging. Upon extraction, the osteosynthesis devices were washed with phosphate-buffered saline (PBS) and incubated with vancomycin-IRDye800CW (vanco-800CW; 0.14 nmol mL^−1^; Li-COR Biosciences, NE, USA) for 15 min at 37 °C. Subsequently, the incubated devices were washed twice with PBS to remove unbound vanco-800CW and imaged in the near-infrared range with an IVIS Lumina II imaging system (excitation 710 nm, emission filter ICG, exposure times 1–10 s; PerkinElmer Inc., USA) and an intraoperative Explorer Air camera coupled to a closed-field imaging box (Vault; excitation 760 nm, emission filter Semrock FF01-819/44–25, exposure times 100–200 ms; SurgVision B.V. Groningen, NL). To assess the autofluorescence of the extracted device, it was imaged prior to and post tracer incubation (Amersham Typhoon Biomolecular Imager; Filter IRLong 825BP30). To verify and visualize Gram-positive bacterial biofilms on extracted devices, microscopic imaging was performed with a two-photon confocal laser scanning microscope (objective 5 × /0.16, filters 500–550/575–610, wavelength 850 nm, Zeiss LSM 7MP) using vancomycin-BODIPY FL as a tracer (0.14 nmol mL^−1^; Thermo Fisher Scientific, USA). Of note, the use of a two-photon microscope was necessary due to the shape and size of the materials tested (plates and screws) in order to have enough distance between the sample and objective. In turn, this required the application of vancomycin-BODIPY FL instead of vanco-800CW because our two-photon microscope cannot image fluorescence in the near-infrared range. The fluorescence imaging workflow of extracted osteosynthesis devices is schematically represented in Fig. 1.

### Sonication and culturing of extracted osteosynthesis devices

The presence of microorganisms on the halved extracted plates and screws selected for regular diagnostics (see the aforegoing section) and on collected tissues was independently investigated at the diagnostic microbiology laboratory of the University Medical Center Groningen (UMCG). Sonication of extracted osteosynthesis devices was performed as described by Trampuz et al. [11], with subsequent culturing of the sonicate on aerobic blood agar (BA), chocolate agar, anaerobic BA, and in blood culture bottles (BD BACTEC) as liquid culture. The decision whether osteosynthesis devices and/or tissues were infected (Supplementary Table S1) was taken by the medical team, including a trauma surgeon, infectious disease specialist, and a clinical microbiologist according to the standard protocol [4]. In addition, extracted osteosynthesis devices were replica plated on BA plates to correlate fluorescent signals to the presence of bacteria.

### Vancomycin-BODIPY FL binding to vancomycin-resistant enterococci

Vancomycin-resistant *Enterococcus faecium* (VRE) strains carrying the *vanA* or *vanB* genes, vancomycin sensitive *Enterococcus faecalis*, and the Gram-negative control bacteria *Escherichia coli* and *Klebsiella oxytoca* were grown overnight in 3 mL Tryptic Soy Broth (TSB) at 37 °C under constant agitation (250 RPM). Overnight, cultures were diluted up to an optical density at 600 nm (OD_600_) of 2 in a final volume of 1 mL. The bacteria were harvested by centrifugation for 3 min at 14.000 RPM. Subsequently, the bacteria were resuspended in 300 µL of 0.14 nmol mL^−1^ vancomycin-BODIPY FL (Thermo Fisher Scientific, USA) and incubated for 15 min in the dark at 37 °C. The bacteria were then washed once with PBS to remove any unbound tracer, and 100 µL aliquots of each sample were transferred in triplicate to a flat-bottomed transparent 96-well plate for fluorescence measurements in a Biotek Synergy 2.0 plate reader (BioTek Instruments, Inc., USA). Fluorescence was measured with filters optimal for FITC; the excitation was set at 480 nm and emission at 520 nm, with the optics position at the “bottom.” For control, triplicate samples of the bacteria incubated and washed only in PBS were also included in the plate. All measurements were carried out in triplicate at 37 °C without shaking. In addition, the fluorescence of the same 96-well plate was imaged with an Amersham Typhoon Biomolecular Imager (filter Cy2 525BP20).

### Data analysis and statistics

Macroscopic fluorescence images and mean fluorescence intensity (MFI) values were analyzed using ImageJ (National Institutes of Health, MD, USA) and Living Image 4.7.3 (PerkinElmer Inc., USA) software. Microscopic images were analyzed using Imaris 9.5.0 software. Regions of interest (ROIs) were drawn around the stained osteosynthesis devices after which the fluorescence signal was quantified. The background signal was quantified by drawing ROIs in uncontaminated biomaterials. Mann–Whitney *U* tests were used to determine the statistical significance of differences in fluorescence between infected or noninfected extracted devices and devices that had not been incubated with vanco-800CW. A *p*-value of < 0.05 was considered statistically significant.

### Ethical approval

Only surplus osteosynthesis devices that were considered unnecessary for clinical diagnosis were used for the present bacteria-targeted imaging with vanco-800CW or vancomycin-BODIPY FL in accordance with the permission obtained from the Medical Ethical Review Board of the UMCG (permission number METc 2016/481). All experiments were performed with adherence to the guidelines of the Declaration of Helsinki and local regulations. Patient data were used pseudo-anonymously based on informed consent.

## Results

**Fig. 2:**
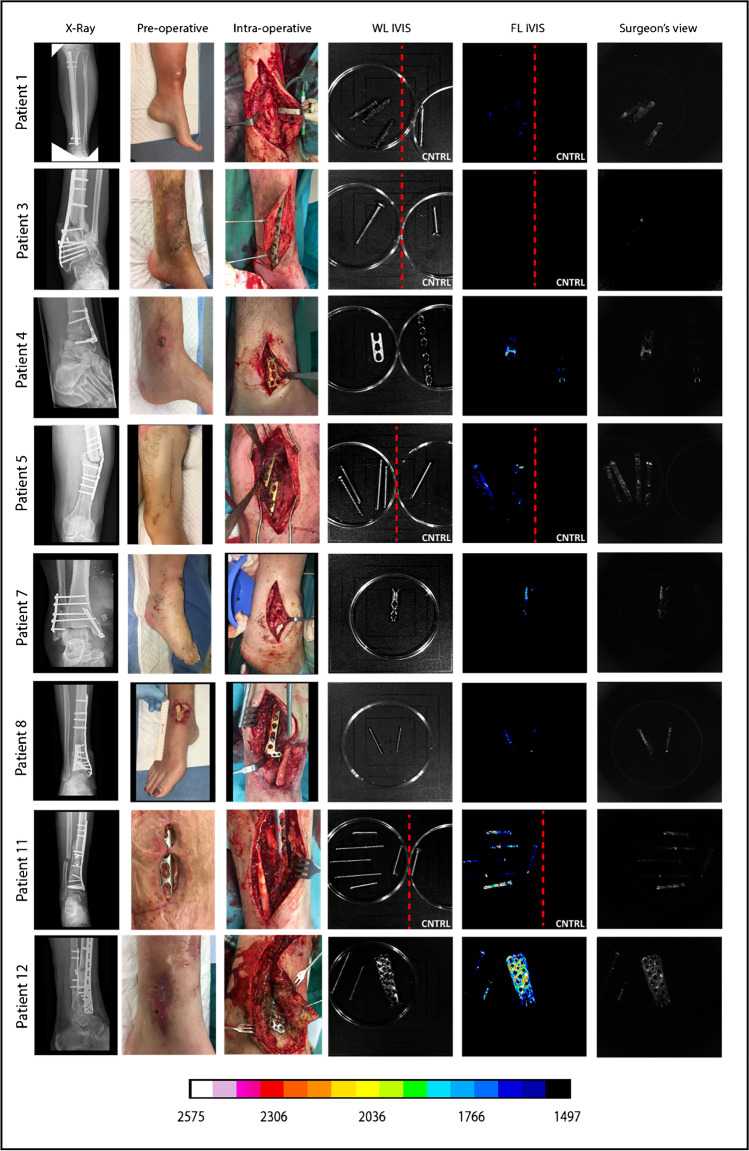
Bacteria-targeted fluorescence imaging of extracted osteosynthesis devices from patients with microbiologically confirmed fracture-related infections. Corresponding X-ray, preoperative, intraoperative, white light (WL IVIS), and fluorescence (FL IVIS) images (IVIS Lumina II imaging system, PerkinElmer Inc., USA; excitation 710 nm; emission filter ICG, exposure time 10 s) of 8 patients and their extracted osteosynthesis devices with a microbiologically confirmed fracture-related infection (FRI) after surgery. The right column shows the raw images as can be viewed by the surgeon (labeled as “Surgeon’s view”). The presence of microorganisms on extracted osteosynthesis devices and surrounding tissue was investigated by culturing in the diagnostic microbiology laboratory of the University Medical Center Groningen, independently from the bacteria-targeted imaging with vanco-800CW. Patient 1, diagnosis: FRI after intramedullary nailing of a crural fracture. Clinical signs: local redness and swelling with elevated inflammation markers (C-reactive protein 347 mg/L). Surgery: removal of the nail, local debridement of the pretibial abscess, and cavitary defect of the tibia. Perioperative cultures: low growth density of *Streptococcus anginosus* (Gram-positive)*.* Imaging: positive, however, weak signal due to a low bacterial density. Patient 3, diagnosis: infected nonunion of the distal tibia with plate breakage. Clinical signs: scar tissue on the medial side of the lower leg with some redness. Surgery: removal of the broken plate and screws, debridement and washout of the nonunion site, temporary antibiotic cement spacer (Masquelet procedure), and application of an external fixator. Perioperative cultures: *Staphylococcus aureus*, *Staphylococcus epidermidis* (Gram-positive), and *Enterobacter cloacae* (Gram-negative). Imaging: positive, however, weak signal due to low Gram-positive bacterial density. Patient 4, diagnosis: FRI after multiple operations of a comminuted distal tibial fracture. Clinical signs: redness and fistula at the medial malleolus. *Surgery*: excision of the fistula, removal of osteosynthesis devices, and local debridement and washout. Perioperative cultures: *Acinetobacter radioresistens* (Gram-negative), *Citrobacter koseri* (Gram-negative), *Corynebacterium aurimucosum* (Gram-positive), *Corynebacterium jeikeium* (Gram-positive), *Dermabacter hominis* (Gram-positive), and *Staphylococcus haemolyticus* (Gram-positive). Imaging: positive. Patient 5, diagnosis: infected nonunion of the left femur with a plate breakage. Clinical signs: no clinical signs of infection at the lateral thigh. Surgery: removal of the osteosynthesis devices, debridement of the nonunion site, and reosteosynthesis with an intramedullary nail. Perioperative cultures: *Cutibacterium* (*Propionibacterium*) *acnes* (Gram-positive). Imaging: positive. Note that these data are also included in Fig. 7. Patient 6, diagnosis: FRI after osteosynthesis of a Gustillo grade 3 complicated ankle fracture. Clinical signs: pain, swelling, redness, septic arthritis, and elevated inflammation markers (CRP 280 mg/L). Surgery: removal of the osteosynthesis devices, washout of the ankle joint, and reosteosynthesis of the medial malleolus. Perioperative cultures: *Staphylococcus aureus* (Gram-positive). Imaging: positive. Patient 8, diagnosis: FRI after osteosynthesis of a distal tibial fracture. Clinical signs: wound dehiscence with the partially exposed plate. Surgery: removal of ventral plate and washout of the wound. Perioperative cultures: *Enterococcus faecalis* (Gram-positive) and *Staphylococcus aureus* (Gram-positive). Imaging: positive. Patient 11, diagnosis: infected nonunion of a crural fracture. Clinical signs: wound dehiscence and exposed implant. Surgery: removal of infected osteosynthesis devices and a washout of the nonunion site. Perioperative cultures: *Staphylococcus aureus* (Gram-positive). Imaging: positive. Note that these data are also included in Fig. 6. Patient 12, diagnosis: infected nonunion of the distal tibia. Clinical signs: local redness and swelling around the scar on the medial malleolus. Surgery: removal of plates and screws, debridement of the nonunion, temporary antibiotic cement spacer (Masquelet procedure), and soft tissue coverage with a latissimus dorsi flap. Perioperative cultures: *Enterococcus faecalis* (Gram-positive), *Staphylococcus capitis* (Gram-positive) and S*taphylococcus epidermidis* (Gram-positive). Imaging: positive. Images were analyzed with ImageJ and Living Image 4.7.3 software

**Fig. 3:**
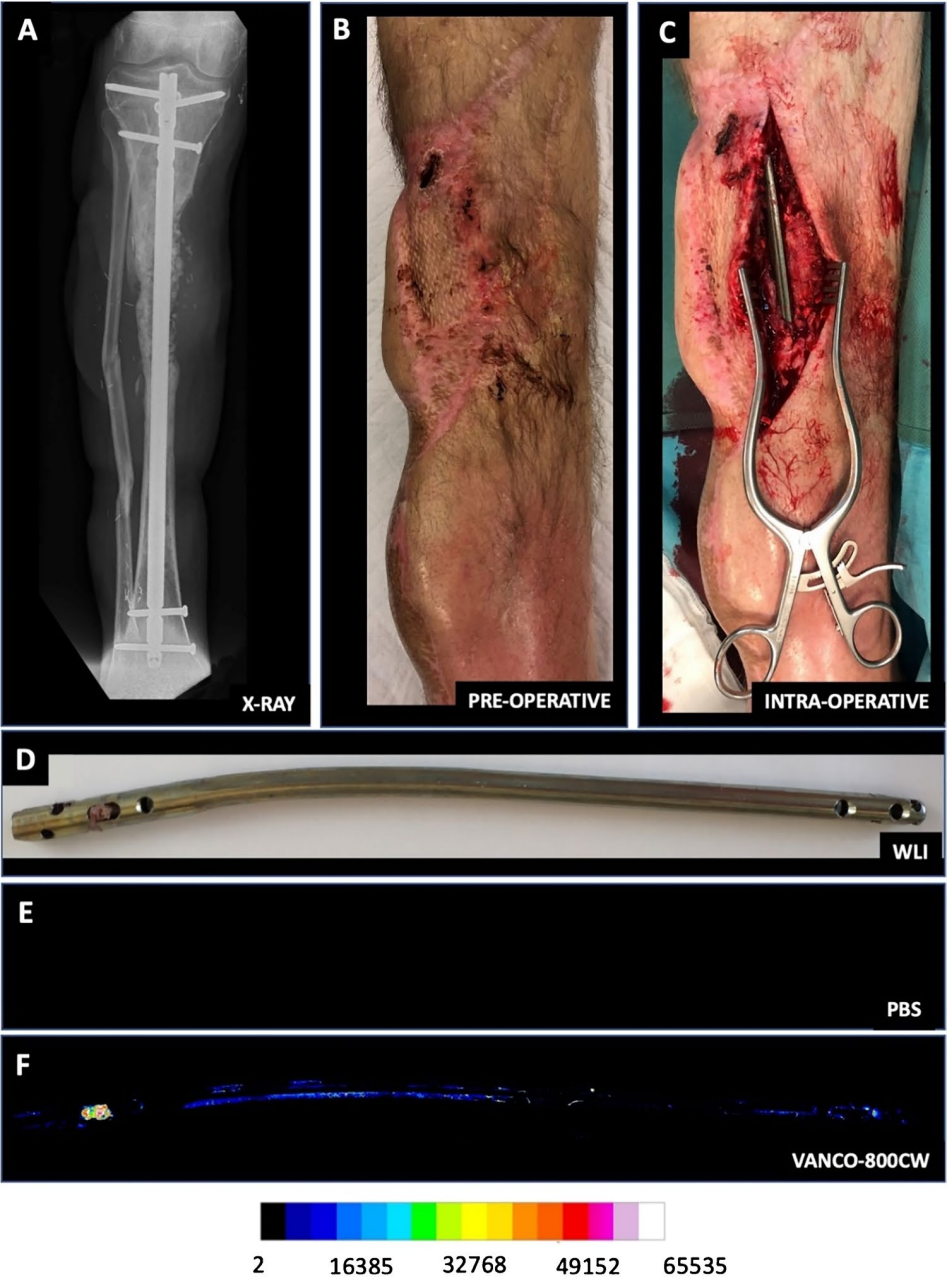
Fluorescence imaging of an extracted tibia nail from a patient with the microbiologically confirmed fracture-related infection prior and post-incubation with vanco-800CW. Patient 13, diagnosis: infected nonunion of the proximal tibia after multiple operations for a complicated crural fracture. Clinical signs: redness, swelling, and fistula proximal tibia. Surgery: removal of the nail, debridement and washout of the nonunion site, and temporary antibiotic cement spacer (Masquelet procedure). Perioperative cultures: *Staphylococcus aureus* (Gram-positive)*.* Imaging: positive. The upper panels show an X-ray image of the patient’s lower leg (A), the preoperative clinical presentation (**B**), and an intraoperative image with the infected tibia nail prior to extraction (**C**). **D** WL image of the extracted tibia nail. **E** Fluorescence image prior incubation with vanco-800CW. **F** Fluorescence image of the tibia nail after 15 min of incubation with vanco-800CW and subsequent washing with PBS. Images were taken with an Amersham Typhoon Biomolecular Imager (Filter IRLong 825BP30) and analyzed with ImageJ software

**Fig. 4:**
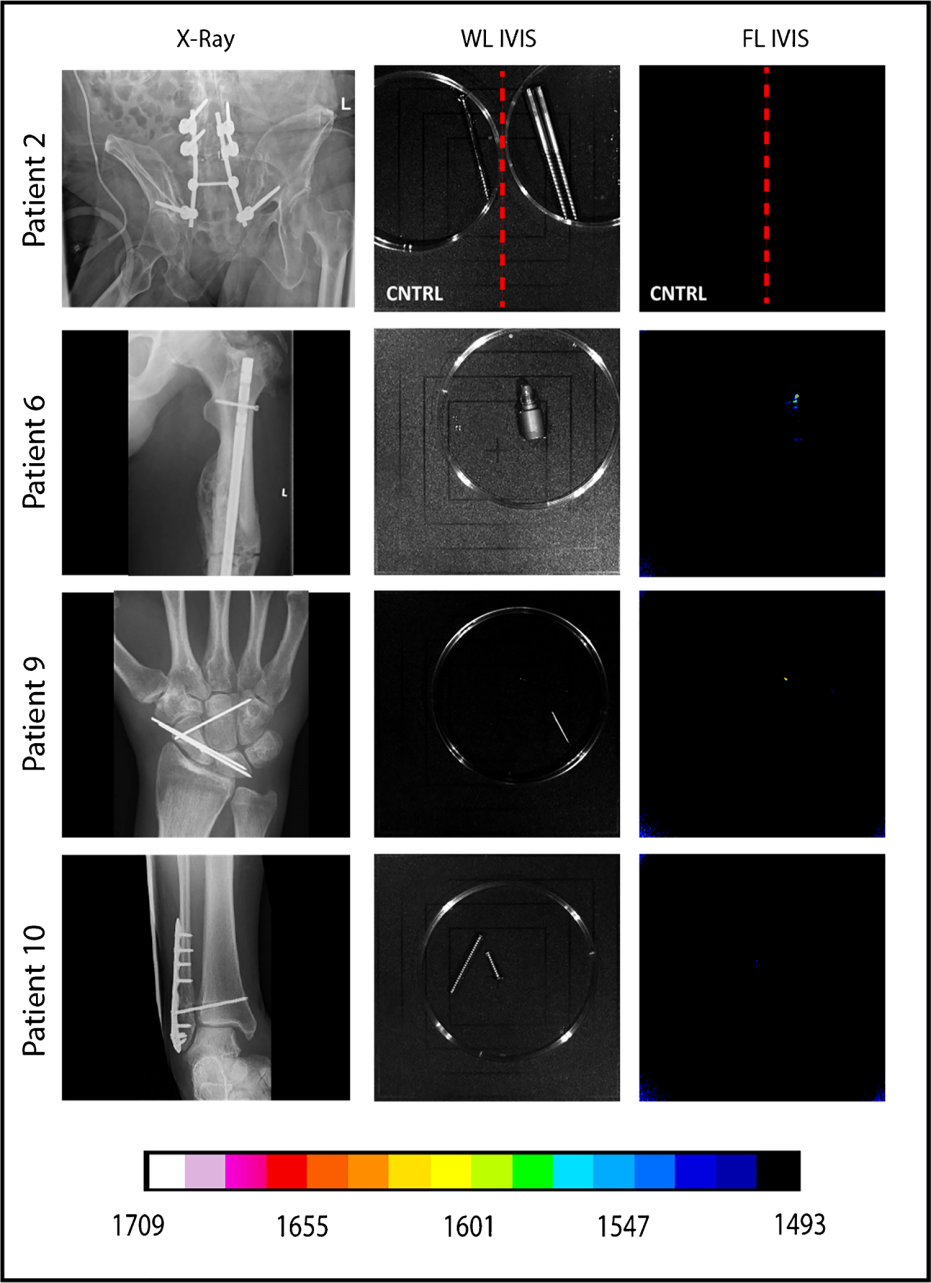
Bacteria-targeted fluorescence imaging of four confirmed culture-negative osteosynthesis devices. Corresponding X-ray, white light (WL IVIS) and fluorescence (FL IVIS) images (IVIS Lumina II imaging system, PerkinElmer Inc., USA; excitation 710 nm; emission filter ICG, exposure time 10 s) of four patients without detectable implant infections. The absence of microorganisms on extracted osteosynthesis devices was concluded based on culturing in the diagnostic microbiology laboratory of our hospital, independently from the bacteria-targeted imaging with vanco-800CW. None of the patients showed clinical symptoms of inflammation, and all imaging results of extracted osteosynthesis devices incubated with vanco-800CW were negative. Patient 2, extracted lumbo-pelvic fixation device due to lower back pain. Patient 6, removed the endcap of a femur nail during exchange nailing for a nonunion of a femoral fracture. Patient 9, routinely removed K-wires of the right wrist after treatment of carpal injuries. Patient 10, routinely removed lag screw after open reduction internal fixation of a Weber C ankle fracture. Images were analyzed with ImageJ and Living Image 4.7.3 software

**Fig. 5:**
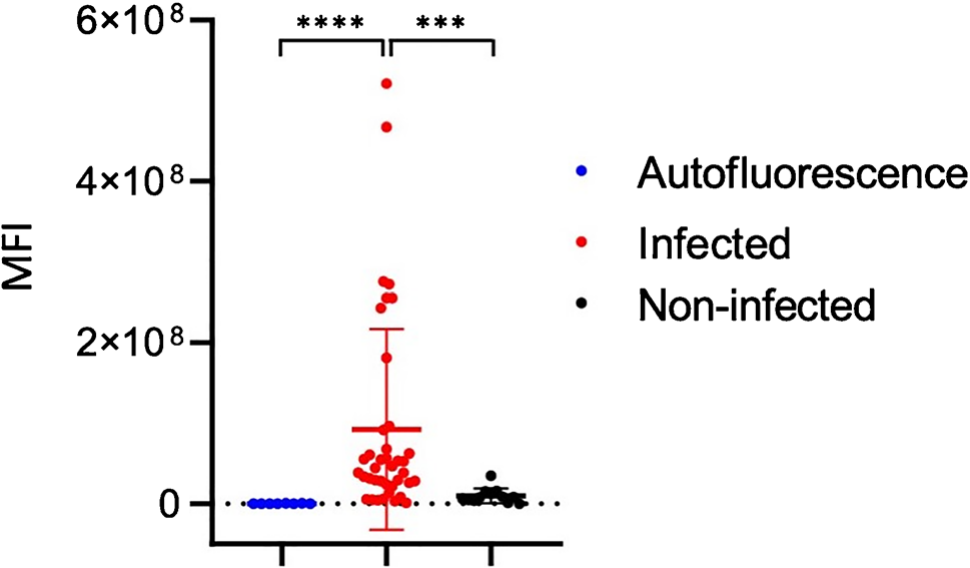
MFI values of all extracted osteosynthesis materials imaged with vanco-800CW. Comparison of the MFI values from all 59 extracted osteosynthesis devices imaged with vanco-800CW shows a significantly increased MFI of materials from patients with an infection (red) compared to materials from patients without infection (black). Moreover, a significant difference was observed between the mean autofluorescence intensity measured in the absence of tracer (blue) and the MFI of infected materials incubated with vanco-800CW. Mann–Whitney *U* tests were used for statistical analyses. Significant differences between groups are marked (****P < 0.0001; ***P = 0.0001). Values represent the mean ± SEM

The investigated patient cohort consisted of 13 patients who underwent revision surgery in the UMCG between January 2018 and February 2020 to extract osteosynthesis devices after a bone fracture treatment. Patients were either operated upon clinical suspicion of FRI (i.e., redness, swelling, persistent wound-leakage, nonunion, plate breakage; *n* = 9; Figs. 2 and 3) or for the removal of osteosynthesis devices for mechanical reasons (*n* = 4; culture-negative controls; Fig. 4). Importantly, the surgically removed plates were cut into two parts. One-half of the plate was used for fluorescence imaging with vanco-800CW, whereas the other half was used for regular diagnostic microbiological culturing (Table S1). Likewise, extracted screws and nails were divided up for either imaging with vanco-800CW or microbiological culturing. Altogether, 59 extracted osteosynthesis devices (i.e., plates, screws, and nails) were treated with vanco-800CW and imaged macroscopically as schematically represented in Fig. 1. To verify and visualize the presence of Gram-positive bacterial biofilms, particular extracted devices were also imaged in triplicate by fluorescence microscopy using vancomycin-BODIPY FL as a tracer. Importantly, evaluation of the imaging results obtained with the extracted osteosynthesis devices from all patients included in the present proof-of-principle study with vanco-800CW allowed a generally clear distinction of infected (Figs. 2 and 3) and noninfected devices (Fig. 4), as was independently verified by microbiological culturing of the other half of the extracted devices (Table S1). Of note, Fig. 2 also presents the raw fluorescence images (labeled as “surgeon’s view”) that can be intraoperatively viewed by the surgeon. Statistical analysis of the imaging results obtained for all 59 extracted osteosynthesis devices treated with vanco-800CW showed significant differences between infected and noninfected devices (Fig. 5). Importantly, the vanco-800CW fluorescence signal could be easily distinguished from light reflections and the autofluorescence of the extracted devices themselves, as illustrated in Fig. 3. The latter observation is consistent with our previous observations that there is no fluorescence signal of bacterial biofilms, human tissues, or osteosynthesis materials detectable in the near-infrared range prior to vanco-800CW administration [9, 10, 12], and this conclusion is fully supported by the absent or marginal fluorescence signals as observed for extracted culture-negative osteosynthesis devices shown in Fig. 4. Importantly, the observed lack of tissue autofluorescence in the 800-nm range is perfectly in line with the known autofluorescence of human tissues, which is lowest in the near-infrared and far-red range of the spectrum [6, 10]. In the following sections, the visualization of FRI by staining extracted osteosynthesis devices with vanco-800CW is documented in detail through two clinical cases of, respectively, acute and low-grade infections.

### Bacteria-targeted imaging in an infected non-union of a tibial fracture

**Fig. 6:**
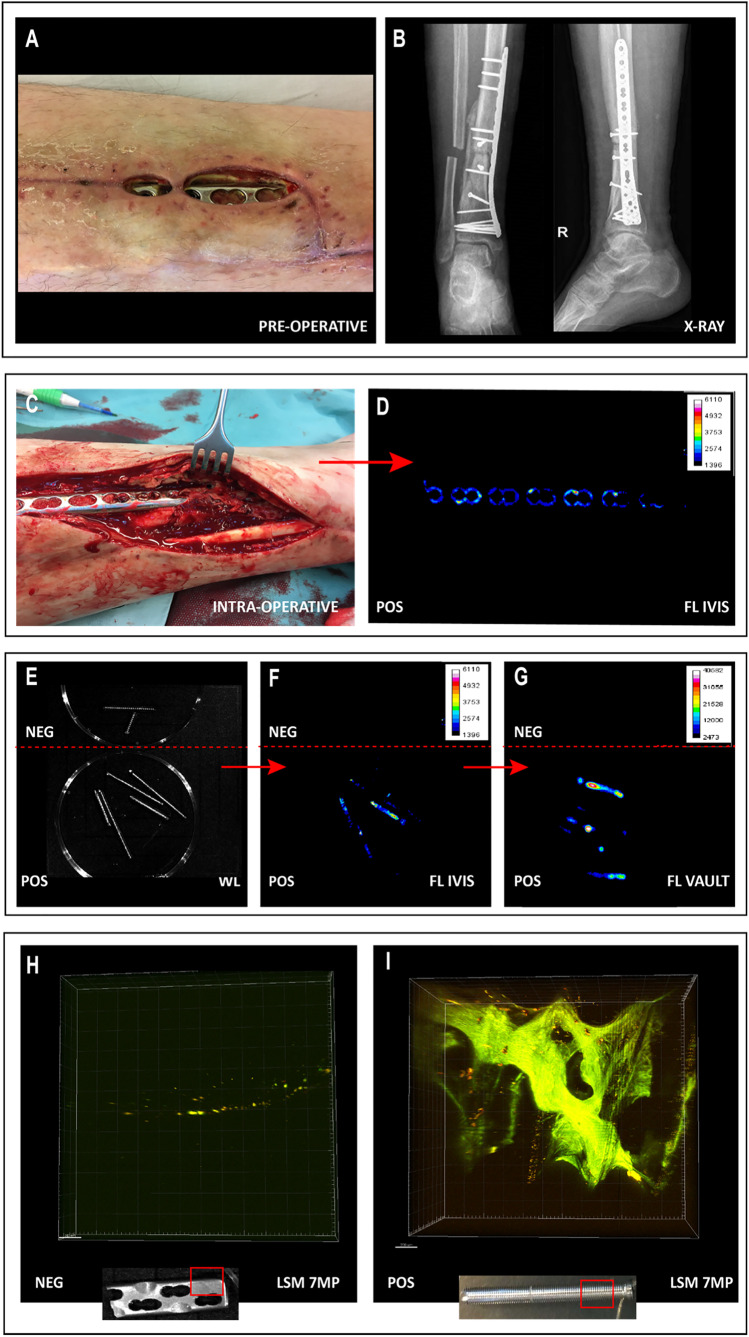
Bacteria-targeted imaging with fluorescently labeled vancomycin of a fracture-related lower leg implant infection. Images relate to patient 11 unless indicated otherwise. **A** Preoperative clinical presentation with wound dehiscence and an exposed plate of the lower leg. **B** X-ray of the delayed union of a crural fracture with tibial plate fixation. **C** Intraoperative image showing the infected tibial plate prior to extraction. **D** Macroscopic fluorescence image (FL IVIS) of the extracted plate upon staining with vanco-800CW. **E** WL image of both infected (POS) and confirmed culture-negative (NEG) extracted screws. Note that the culture-negative screws were obtained from a different patient (patient 10; Fig. 4), but imaged simultaneously with infected screws of patient 11. **F**, **G** Fluorescence images of the screws presented in **E** upon staining with vanco-800CW performed with two different imaging devices, respectively, an IVIS Lumina II (**F** PerkinElmer Inc., USA; excitation 710 nm; emission filter ICG, exposure time 10 s) and an intraoperative camera (**G** Explorer Air coupled to a closed-field imaging box [Vault], SurgVision B.V. Groningen, NL; excitation 760 nm, emission filter Semrock FF01-819/44–25 nm, exposure times 100–200 ms). Of note, there is a slight mismatch in the fluorescence signals in the images of panels **F** and **G**, which relates to slightly different positions of the imaged screws in the IVIS and the intraoperative camera system. Further, different settings were applied in the two systems (i.e., a binning of 4 in the IVIS and a binning of 1 in the Vault), which explains the differences in pixel size. **H**, **I**. Microscopic image of negative control (**H**) and infected screw (**I**) upon staining with vancomycin-BODIPY FL showing the biofilm dimensions. Microscopy was performed with a two-photon confocal laser scanning microscope (Zeiss LSM 7MP). Images were analyzed with ImageJ, Living Image 4.7.3, and Imaris 9.5.0 software

A first clinical example of successful bacteria-targeted fluorescence imaging with vanco-800CW in a clinical setting was obtained for a patient with tibial nonunion accompanied with acute FRI (Fig. 6). In this case, a 51-year-old man (patient 11) suffered a lower leg fracture for which he was initially treated with a cast. After 7 months, barely any fracture healing of the tibia had occurred and therefore operative debridement of the nonunion site, followed by a cancellous bone graft and tibial plate fixation was performed. The postoperative course was complicated by FRI, characterized by wound dehiscence and eventually an exposed implant (Fig. 6A, B). Therefore, the infected plate and screws had to be removed. The plate was surrounded by debris and minimal cloudy seroma (Fig. 6C).

Figures 6C and D, respectively, show the tibial plate in situ and upon macroscopic analysis after extraction and 15 min incubation with vanco-800CW. The latter revealed a clear fluorescence signal with a patchy distribution (Fig. 6D), which was also the case for the extracted screws (Figs. 6E–G, marked “POS”). In contrast, control osteosynthesis devices of the confirmed infection-negative patient 10 emitted no fluorescence signals (Figs. 6E–G, marked “NEG”). To verify and visualize the presence of Gram-positive bacterial biofilms, extracted screws were stained with vancomycin-BODIPY FL and imaged with a two-photon microscope. Indeed, biofilm formation was clearly visualized, demonstrating the high binding specificity of vancomycin-based tracers (Fig. 6H, I; for video, see Supplementary Movie S1). Sonication of extracted biomaterials and subsequent microbiological culturing identified the Gram-positive bacterium *S. aureus* as the causative agent of the infection (Table S1).

### Detection of low-grade FRI with vanco-800CW

**Fig. 7:**
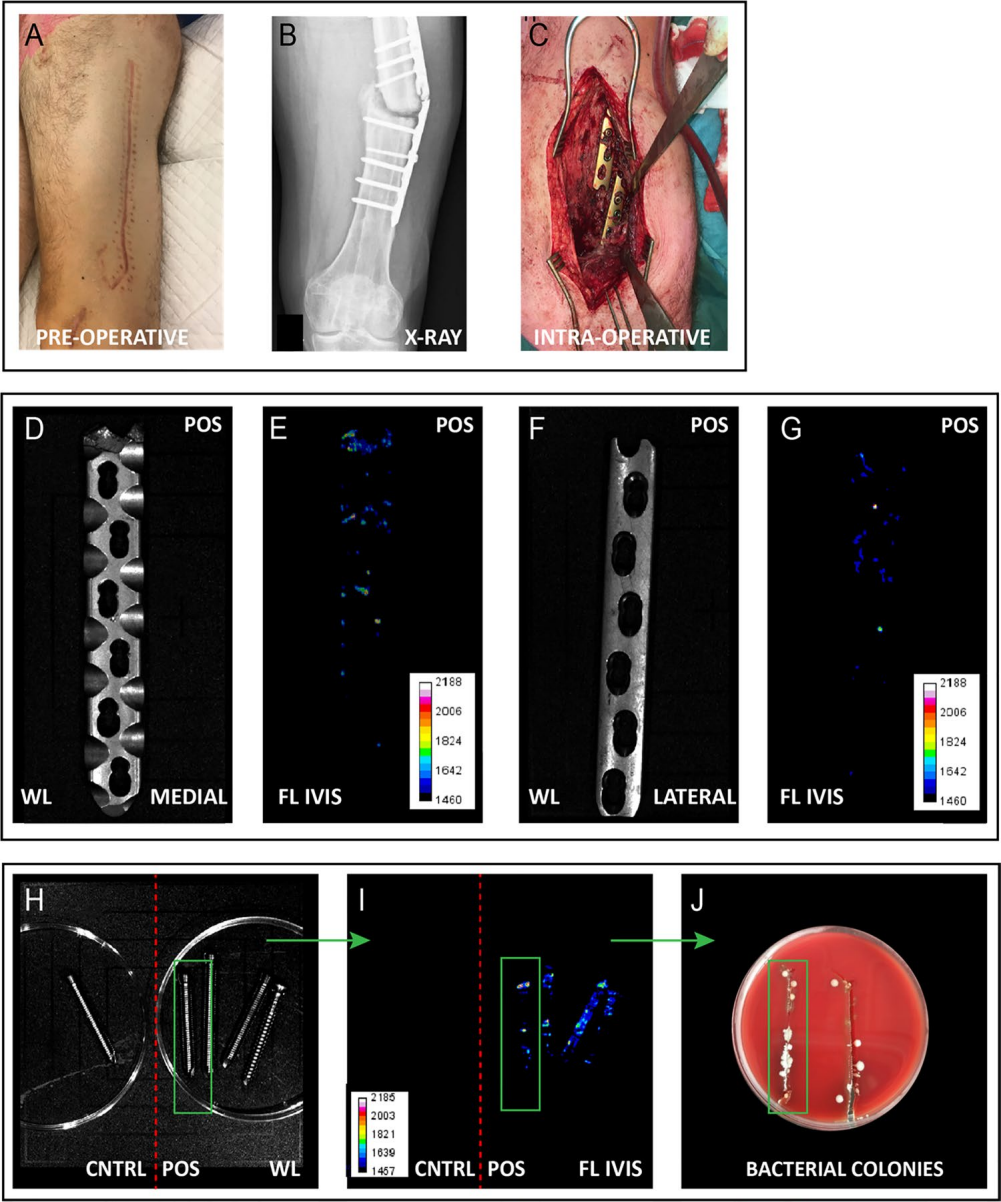
Bacteria-targeted fluorescence imaging and corresponding microbiological culturing of a fracture-related femur infection. Images relate to patient 5. **A** Preoperative clinical presentation with no signs of infection at the lateral thigh. **B** X-ray of a nonunion of a femoral fracture with a broken plate. **C** Intraoperative image showing no signs of infection (e.g., no fluid, abscesses, or diseased bone around the broken implant). **D–G** Macroscopic WL images of the back and front sides of the extracted plate (**D**, **F**) with corresponding fluorescence images (**E**, **G**. IVIS Lumina II; excitation 710 nm; emission filter ICG, exposure time 10 s). **H**, **I** WL image (**H**) and corresponding fluorescence image (**I**) of suspected infected (POS) and new negative control (CNTRL) screws (IVIS Lumina II; excitation 710 nm; emission filter ICG, exposure time 10 s). **I**, **J** Correlation between fluorescence images (**I**) with bacterial colonies on a blood agar plate (**J**). Images were analyzed with ImageJ and Living Image 4.7.3 software

The high sensitivity and specificity of bacteria-targeted optical imaging with vanco-800CW are supported by a second case, where a patient with a fracture-related low-grade infection of the femur presented no clinical symptoms of infection prior to surgery (patient 5; Fig. 7A). The X-ray showed a nonunion of a femoral fracture with a broken plate (Fig. 7B). During surgery, no signs of bacterial infection were observed (Fig. 7C, D, F). Nonetheless, relatively low but clear fluorescence signals were detectable upon 15 min incubation of the extracted plate and screws with vanco-800CW (Fig. 7E, G, I). Moreover, the fluorescence signals were directly correlated to bacterial growth upon replica plating of the extracted screws on BA plates (Fig. 7H–J). Microbiological culturing revealed that osteosynthesis devices of this patient carried the Gram-positive bacterium *Cutibacterium* (*Propionibacterium*) *acnes* (Table S1).

### Detection of vancomycin-resistant Gram-positive bacteria

**Fig. 8:**
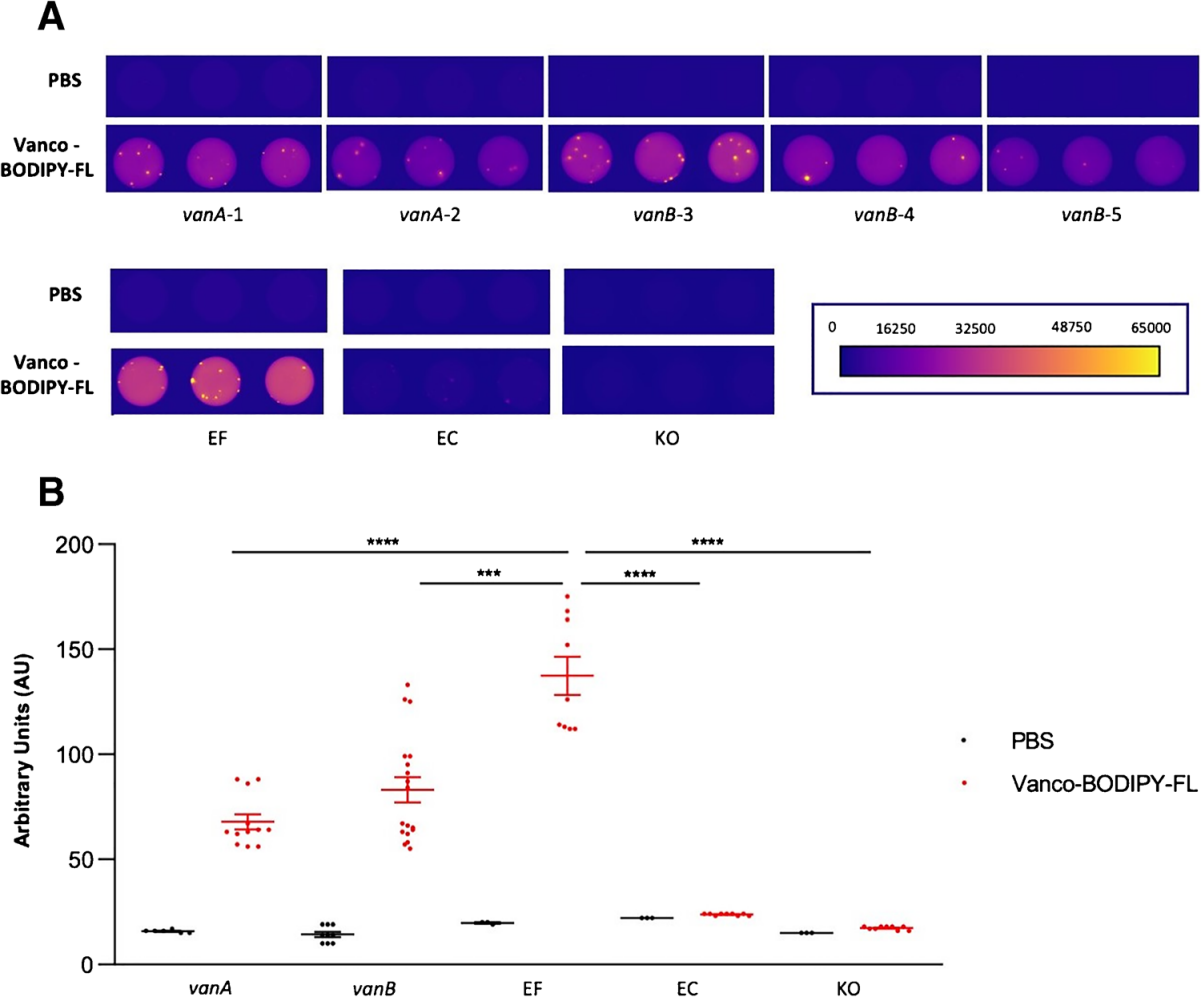
Binding of vancomycin-BODIPY FL by vancomycin-resistant Gram-positive bacteria. **A** Macroscopic imaging of *vanA-* or *vanB-*positive vancomycin-resistant clinical *E. faecium* isolates (Gram-positive), a vancomycin sensitive clinical *E. faecalis* isolate (EF; Gram-positive), *E. coli* (EC; Gram-negative), and *Klebsiella oxytoca* (KO; Gram-negative) upon incubation with vancomycin-BODIPY FL and subsequent washing with PBS. Of note, the *vanA*-1 and *vanB*-5 strains still test sensitive for vancomycin with MIC values ≤ 0.5 mg.L^−1^, whereas the *vanA*-2, *vanB*-3, and *vanB*-4 strains test resistant for vancomycin with MIC values of 8, ≥ 32, and 8 mg.L^−1^, respectively, as analyzed by Vitek2 (bioMérieux). For control, PBS-washed bacteria were imaged that had not been incubated with vancomycin-BODIPY FL. Images were recorded with an Amersham Typhoon Biomolecular Imager (Filter Cy2 525BP20). **B** Quantification of the vancomycin-BODIPY FL-specific fluorescence of the bacteria imaged in **A** in arbitrary units. The red data points mark the measurements for bacteria labeled with vancomycin-BODIPY FL, while the black data points mark controls for autofluorescence of the bacteria that were only washed with PBS. Fluorescence was measured in a Biotek Synergy 2.0 plate reader (BioTek Instruments, Inc., USA; filters optimal for FITC; excitation was set at 480 nm and emission at 520 nm, with optics position at the “bottom”). Mann–Whitney *U* tests were used for statistical analyses. Significant differences between groups are marked (****P < 0.0001; ***P = 0.0002). Values represent the mean ± SEM

Lastly, to investigate whether the use of vancomycin-based tracers could be limited by increased vancomycin resistance in Gram-positive bacteria, we assessed the binding of vancomycin-BODIPY FL to clinical *vanA-* or *vanB*-positive *E. faecium* isolates that are resistant to vancomycin (VRE) and a vancomycin-sensitive *E. faecalis* control isolate. In addition, we used the Gram-negative bacteria *E. coli* and *K. oxytoca* as controls. As shown in Fig. 8, the VRE isolates bound significantly more vancomycin-BODIPY FL than the Gram-negative control strains, although the binding of this tracer by the VRE isolates was reduced compared to the vancomycin-sensitive *E. faecalis* isolate. Altogether, these observations imply that vancomycin-based optical tracers do allow the detection of vancomycin-resistant Gram-positive bacteria.

## Discussion

The present clinical proof-of-principle study indicates that detection of bacterial growth on osteosynthesis devices by optical imaging with vanco-800CW is highly specific, sensitive, fast, and feasible in the clinical setting. On the contrary, the current diagnosis of FRI relies heavily on culture-based methods that may take several days. Hence, patients with suspected FRI are often treated empirically with revision surgery and broad-spectrum antibiotics until the causative organism(s) have been identified. Here, we show that imaging of potentially infected osteosynthesis devices with vanco-800CW reduces the time-to-result from days to less than 30 min. This implies that bacteria-targeted fluorescence imaging may significantly enhance intraoperative clinical decision-making regarding empirical antibiotic treatment, extensive debridement, the choice between one- and two-stage revision surgery, or implant exchange [13]. A possible limitation of the fluorescence imaging approach with vanco-800CW is that it cannot replace current diagnostic procedures because it does not identify the causative agents of FRI and the associated antibiotic resistance. Hence, we must regard optical imaging with vanco-800CW as a complementary tool to the current diagnostic methods for prompt visualization of an infection. Ideally, a definitive diagnosis of the causative agent is needed to direct antibiotic treatment, which may be achieved in the future by the use of multiple tracers next to vanco-800CW, i.e., targeting Gram-negative bacteria or specific bacterial species.

Based on the present results, we envisage that intraoperative topical application of vanco-800CW, incubation and rinsing off excess and unbound tracer during surgery may provide surgeons with real-time visual information on the presence and extent of infection. Here, it is noteworthy that imaging can be performed with doses of vanco-800CW that are ~ 20 to 40-fold below the minimal inhibitory concentration (MIC) of vancomycin for staphylococci (https://eucast.org/clinical_breakpoints/) [8, 10]. Thus, the results of routine diagnostic procedures and culturing will not be affected by the imaging procedure. Another clear advantage of vanco-800CW is its fluorescence in the near-infrared range, which minimizes interference by tissue autofluorescence [6, 10, 14, 15]. In our study, an IVIS imaging system and an intraoperative camera coupled to a closed-field imaging box were used to detect the emitted fluorescence signal. The latter camera system is already in use for surgical applications in the operating theater [16, 17]. Yet, prior to any intraoperative application of vanco-800CW, it will be necessary to assess its safety, although vancomycin [18] and the IRDye800CW fluorophore have separately been approved for clinical implementation [19, 20]. A second limitation of vanco-800CW is that it will probably not detect FRI caused by Gram-negative bacteria, as exemplified in the present study by the lack of vancomycin-BODIPY FL binding to *E. coli* and *K. oxytoca*. This is in accordance with the fact that vancomycin shows the highest specificity for Gram-positive bacterial cell walls. However, Gram-negative bacteria cause ~ 30% of all cases [21], which implies that vanco-800CW will allow a correct diagnosis of FRI in most cases. This view is supported by the present results and, in particular, by the observation that even notoriously vancomycin-resistant bacteria, such as VRE, can bind vancomycin-based tracers.

Both from the diagnostic and scientific perspectives, it would be interesting if the fluorescent signals detected with tracers like vanco-800CW could be used to quantify the numbers of viable bacteria on extracted osteosynthesis devices and to determine the cutoff lines for positive testing of infection. However, the quantification of viable bacteria on extracted implants based on the vanco-800CW fluorescence signal is technically challenging. In the first place, different Gram-positive bacterial species that cause implant-associated infections will bind vancomycin with different efficiencies, and this also applies to vanco-800CW as we have previously measured in vitro for *S. aureus*, *S. epidermidis*, Corynebacteria, and various streptococci [9, 10]. This view is fully supported by our present measurements using different VRE isolates (Fig. 8). Hence, as long as the causative infecting organism has not been identified, it is hard to correlate vanco-800CW-specific fluorescence to the numbers of bacterial colony-forming units (CFUs). This becomes even more challenging in the case of polymicrobial infections that are frequently encountered in clinical settings, as underscored by the cases portrayed in Figs. 2 and 3 and the microbiological data in Table S1. In such polymicrobial biofilms, the relative numbers of different bacterial species may vary substantially. Secondly, vanco-800CW will also bind to the cell walls of dead bacteria, which adds to the challenge of correlating fluorescence to CFUs in a clinical setting [9], especially since patients are sometimes receiving antibiotics prior to revision surgery, e.g., in case of systemic infection (Table S1). Of note, while the antimicrobial therapy will decrease the CFU counts, the binding of vanco-800CW to dead bacteria will actually contribute to the sensitivity of our approach in detecting infection on osteosynthesis devices. A third hurdle relates to the fact that bacterial biofilms are not homogeneously distributed over an explanted osteosynthesis device (see Figs. 2 and 3), whereas a part of the explanted device needs to be used for routine clinical diagnostics analyses to specify the causative microorganisms and their antibiotic resistances. The latter is of prime importance with respect to patient care and associated ethical considerations. Accordingly, for the present experimental study, upon surgery extracted osteosynthesis devices had to be divided and separately investigated with priority for clinical diagnostic testing, including sonication and subsequent microbiological culturing. However, this approach is semiquantitative, and the results presented in Table S1 validate, but do not quantify, the detected bacteria on samples that were used for imaging with vanco-800CW.

A potential limitation of our present study is that it is not yet possible to determine a precise receiver operating characteristic (ROC) curve that describes the performance of bacteria-targeted fluorescence imaging with vanco-800CW in the detection of osteosynthesis device infection because there is currently no existing gold standard to compare our results with. In particular, there is currently no gold standard reference to count individual living bacterial cells on osteosynthesis devices and to determine the true positive, true negative, false positive, and false-negative results for a ROC analysis. Also, there is currently no methodology to precisely quantify the in vivo diagnostic accuracy without interference with the diagnostic routine needed for optimal patient care. We anticipate, however, that a ROC can be approximated once larger numbers of clinical samples have been systematically analyzed in a standardized manner. This will be most relevant in the case of low-grade infections that present a very low burden of biofilm. Here, the distinction of bacteria-specific vanco-800CW fluorescence signals can be challenging as may also be the case for routine diagnostic culturing. This is exemplified by extracted osteosynthesis materials from patients 3 and 6. Patient 3 was diagnosed with low-grade infection and the extracted screw from this patient showed a weak fluorescence signal upon incubation with vanco-800CW (Fig. 2). In contrast, extracted osteosynthesis material from patient 6 was confirmed culture-negative but showed a comparably weak fluorescence signal upon incubation with vanco-800CW as the extracted screw from patient 3 (Figs. 2 and 4). We explain the weak signal observed for patient 3 by the fact that this patient presented a low-grade infection of the distal tibia that was associated with *S. epidermidis* growth only upon sonication of the extracted materials (Table S1). Prior to surgery, patient 3’s inflammatory markers were low and during surgery, there were no evident signs of infection. Accordingly, we believe that the low fluorescence signal relates to a very low bacterial density on the imaged screw that was extracted from patient 3. Here, it is important to bear in mind that we had to divide up the extracted materials with priority for clinical microbiological diagnostics. Thus, it is conceivable that the extracted osteosynthesis materials used for clinical microbiological diagnostics might have shown stronger signals upon incubation with vanco-800CW, but this could no longer be tested after sonication of the respective plate and screws. Likewise, we can retrospectively only speculate why a low-intensity signal was detected on the endcap of the extracted femur nail from patient 6. Firstly, patient 6 demonstrated no signs of infection during surgery and, accordingly, the patient was not treated with antibiotics. Since the extracted osteosynthesis materials from patient 6 tested culture-negative, we consider it unlikely that the observed low-intensity fluorescence signal related to a bacterial biofilm. However, we cannot exclude the possibility that a low-grade infection by bacteria that are hard to culture or unculturable has escaped detection, in which case we would be dealing with a false negative culture result. Another possible explanation could be that the washing with PBS to remove unbound tracer after incubation with vanco-800CW was incomplete, in which case we would be dealing with a false-positive result. Importantly, despite some possible ambiguity in the imaging of the extracted osteosynthesis material from patient 6, our present results already show that the detection of osteosynthesis device infections with vanco-800CW has a clear added value in complementing the existing diagnostic routine because (1) it clearly allowed the distinction of infected and noninfected devices; (2) it visualized bacteria on extracted devices in less than 30 min; and (3) it provides an opportunity for rapidly informed decision-taking concerning the further treatment of patients during surgery. Moreover, the collected images have provided unique insights into the distribution of bacterial biofilms on infected osteosynthesis materials in patients.

Lastly, our present study represents the first successful clinical example of bacteria-targeted fluorescence imaging for the rapid visualization of fracture-related infections, even in the case of low-grade infections. This is an area of molecular imaging that is regarded as highly promising but presently still in its infancy. From the clinical microbiological and trauma surgical points of view, we consider it most notable that our proof-of-principle study provides clear yes/no answers to the critical clinical question of whether or not an infection is present. In addition, our findings suggest that bacteria-targeted imaging may serve as a highly sensitive tool for rapid detection of implant infections, ranging from osteosynthesis devices to surgical meshes, stents, prosthetic valves, and vascular grafts. While the present study was exclusively based on the use of fluorescent tracers for the optical imaging of infection, we anticipate that future applications will involve also radioactively labeled bacteria-targeted tracers for the noninvasive diagnosis of FRI and other deep-seated implant infections [5–7, 22, 23].

## Supplementary Information

Below is the link to the electronic supplementary material.

Supplementary file1 (XLSX 12 KB)

Supplementary file2 (AVI 39266 KB)

## Data Availability

All data are provided with the manuscript, except data that identify the included patients. Vanco-800CW was purchased from Li-COR Biosciences, NE, USA.
